# Cocaine use disorder, mental health diagnoses, and serious mental illness characteristics in mental health treatment

**DOI:** 10.1371/journal.pmen.0000337

**Published:** 2026-01-22

**Authors:** Orrin D. Ware, G. Rose Geiger, Monique T. Cano

**Affiliations:** 1 University of North Carolina at Chapel Hill, School of Social Work, Chapel Hill, North Carolina, United States of America; 2 Yale University, School of Medicine, Department of Psychiatry, New Haven, Connecticut, United States of America; University of Western Australia, AUSTRALIA

## Abstract

Over half of treatment-seeking individuals with a cocaine use disorder have a co-occurring mental health disorder. Mental health disorder symptoms may impact an individual’s functioning so severely that they are classified as a serious mental illness (SMI). However, there is variability in defining SMI, including the duration of symptoms, functional impairment, or specific diagnoses such as schizophrenia. This study answered the following questions among a national sample of adults with cocaine use disorder who received services from mental health treatment facilities from 2013 to 2021 (N = 359,500): [1] what are most diagnosed mental health disorders, [2] what is the percentage of SMI, and [3] are some mental health diagnoses associated with the designation of SMI? Binary logistic regression identified mental health diagnoses associated with SMI. The sample was primarily male (n = 184,312; 51.3%) and the largest racial group was Black or African American (n = 149,080; 41.5%). The most endorsed mental health disorder diagnoses in the sample include depressive disorders (n = 115,347; 32.1%), bipolar disorders (n = 94,591; 26.3%), and schizophrenia or other psychotic disorders (n = 86,298; 24.0%). Anxiety disorders, bipolar disorders, conduct disorders, dementia/delirium, depressive disorders, personality disorders, pervasive developmental disorders, schizophrenia or other psychotic disorders, trauma-or stressor-related disorders, and other mental health disorders were associated with SMI. Diagnoses most associated with SMI were schizophrenia or other psychotic disorders (Adjusted odds ratio = 20.188; 95% Confidence Interval = 19.533, 20.865), bipolar disorders (Adjusted odds ratio = 7.257; 95% Confidence Interval = 7.085, 7.433), and depressive disorders (Adjusted odds ratio = 4.855; 95% Confidence Interval = 4.754, 4.957). This study provides a snapshot of the recent landscape of mental health disorders and SMI among individuals with a cocaine use disorder who received treatment from mental health facilities from 2013 to 2021. Significant comorbidity was identified, including multiple diagnoses and SMI.

## Introduction

Approximately 2.8 million adults in the United States used cocaine in 2021, of which almost half (approximately 1.35 million) had a cocaine use disorder [1]. Cocaine use disorder is a prominent public health concern associated with an increased risk of morbidity and mortality [2–4]. Inherent in the diagnostic criteria of a cocaine use disorder, the condition can have a severe impact on an individual’s life, such as damaging interpersonal relationships and causing withdrawal symptoms if someone suddenly stops using the substance [5]. Cognitive deficits may develop because of cocaine use disorder, potentially affecting attention and memory [3,6]. Compared to the general population, crack cocaine use is linked to cardiovascular risk factors and sexually transmitted diseases and infections [2]. Smoking crack cocaine can also result in death due to the rapid absorption of cocaine when it is smoked [2]. Furthermore, using cocaine with other substances increases the potential risk of a fatal overdose [3]. Cocaine-related overdoses, often implicated with the co-use of other substances such as opioids, have increased by over 50%, from 15,883 in 2019–24,486 in 2021 [7,8]. The prevalence of and harmful factors associated with cocaine use disorder, especially co-occurring mental health disorders, indicate its prominence as a public health concern.

Cocaine use disorder and mental health disorders--such as anxiety and depressive disorders--often co-occur [3,9–14]. Lifetime cocaine use disorder prevalence among individuals with a mood disorder is 6.5% and 5.4% among individuals with an anxiety disorder [12]. Among persons with cocaine use disorder seeking treatment, estimates of co-occurring mental health disorders (excluding other substance use disorders) range from 65% to 73% [3,15,16]. Mental health disorders vary by severity, such as the number and intensity of symptoms experienced [5], with the designation of a serious mental illness (SMI) being applied when the impact of diagnosed mental health disorder(s) on an individual’s life is severe. According to the National Institutes of Mental Health and the Substance Abuse and Mental Health Services Administration (SAMHSA), an SMI is defined as a mental health disorder with symptoms so severe that functioning in major life activities is seriously impaired [1,17], with approximately 14 million adults in the U.S. having an SMI based on this definition [1]. However, the definition of SMI varies, with some studies defining it based on duration, others describing it as disability or functional impairment (similar to SAMHSA’s definition), or using specific diagnoses [18,19]. For example, when using duration in the operational definition of SMI, some studies have described the length of time that mental health symptoms were experienced [18,19]. Regarding specific diagnoses, schizophrenia spectrum and other psychotic disorders are also often used as definitions of SMI [18,19]. Although the concept of SMI is not consistent [18,19], individuals are identified as having an SMI in real-world clinical settings.

Despite the association between cocaine use disorder and mental health disorders, there is a gap in the literature regarding the prevalence of mental health disorders and SMI among a real-world national sample of adults with a cocaine use disorder receiving services from mental health treatment facilities. A study examining substance preference among individuals with an SMI who received care from a community treatment team found cocaine to be the second most preferred substance behind alcohol [20]. Considering the variability of how SMI is defined [18], it is necessary to examine whether specific mental health disorder diagnoses are associated with SMI among individuals with a cocaine use disorder. To fill this gap in the literature, we aimed to answer the following questions among a national sample of adults with cocaine use disorder receiving treatment from mental health treatment facilities from 2013 to 2021: [1] what are most commonly diagnosed mental health disorders, [2] what is the percentage of SMI, and [3] are some mental health diagnoses associated with an SMI in this sample?

## Materials and methods

### Dataset

To conduct this study, we used the publicly available SAMHSA-provided Mental Health Client Level Data (MH-CLD) [21]. The dataset contains sociodemographic and clinical characteristics of individuals who received mental health treatment from a facility that reported data to a state or other government body in the United States. As an annual cross-sectional dataset, the MH-CLD provides annual data releases. To ensure the most robust sample was included, we incorporated each year of data available during the time of conducting this study (downloaded April 2, 2024), including 2013–2021.

### Sample selection

The following sample selection criteria were used to identify the sample: (a) received treatment in the US (territories were excluded), (b) not missing data for the primary diagnosis, (c) being at least 18 years old, and (d) have a cocaine use disorder (listed as cocaine abuse or cocaine dependence in the dataset). After applying this sample selection criterion, we retained a sample of 359,500 individuals representing all fifty states and the District of Columbia. As the study focused on the presence of mental health disorder diagnoses and SMI, we did not exclude cases for missing data for the demographic characteristics, as doing so would reduce the sample size by approximately 47%.

### Measures

Variables in the current study fell into three distinct categories: sociodemographic characteristics, mental health disorder diagnoses, and SMI. Sociodemographic characteristics were categorical and included [a] year of treatment, [b] age in years, [c] gender, [d] educational level, [e] living arrangements, [f] marital status, and [g] race and ethnicity. All were captured from the MH-CLD 2013–2021.

#### Mental health disorder diagnoses.

The types of mental health disorder diagnoses were flagged to indicate if an individual had specific diagnoses as their primary, secondary, or tertiary diagnoses [21]. The dataset contains a maximum of three diagnoses per case. The following mental health disorder groupings were flagged with ‘yes’ or ‘no’ by separate variables in the MH-CLD: (a) anxiety disorder diagnosis, (b) ADHD diagnosis, (c) bipolar disorder diagnosis, (d) conduct disorder diagnosis, (e) delirium/ dementia diagnosis, (f) depressive disorder diagnosis, (g) oppositional defiant disorder diagnosis, (h) personality disorder diagnosis, (i) pervasive developmental disorder diagnosis, (j) schizophrenia or other psychotic disorder diagnosis, (k) trauma-or stressor-related disorder diagnosis, and (l) other mental health disorders.

#### Serious Mental Illness (SMI).

SMI is a two-level categorical variable found in the dataset that describes whether the individual has an SMI based on the definition of the state in which they received treatment, with ‘yes’ and ‘no’ as potential values [21].

### Data analysis

All statistical analyses, which include descriptive statistics (such as percentages) and binary logistic regression models, were completed using IBM SPSS Statistics Version 28.0 [22]. The logistic regression models included SMI as the dependent variable and each of the twelve mental health disorder diagnoses as independent variables. Unadjusted models, which examined each of the independent variables separately, were conducted. An adjusted logistic regression model was also completed in which all independent variables were added simultaneously. Each reference group was “no”, indicating that a disorder was not diagnosed among a specific case. As this study focused on whether specific mental health diagnoses were associated with SMI, sociodemographic characteristics were used descriptively and not included in the models. Alongside the associations between specific mental health diagnoses and the presence of SMI, being this study’s focus, 47% of the sample was missing data for sociodemographic characteristics. This would result in nearly half of the cases being excluded if the analyses included the sociodemographic characteristics, as a missing value analysis was conducted and identified the data to be missing completely at random [23,24]. Using the package “ggplot2” [25] in R [26], we created bar charts and a line graph. All study procedures were identified as non human subjects research based on ethical review by the University of North Carolina at Chapel Hill Institutional Review Board.

## Results

### Sample characteristics

Characteristics of the sample may be found in Table 1. Some of the most endorsed factors of the sample include being male (n = 184,312; 51.3%) and having one mental health diagnosis (n = 181,655; 50.0%). The largest age group in the sample was 50–59 years old (n = 113,521; 31.6%). Clinical settings in which the cases received treatment included community-based programs (n = 346,783; 96.5%), institutions in the justice system (n = 10,768; 3.0%), psychiatric hospital (n = 17,188; 4.8%), residential treatment (n = 5,286; 1.5%), and other psychiatric inpatient (n = 24,961; 6.9%).

**Table 1:** Sociodemographic Characteristics of the Sample.

Characteristics	Study Sample		Has a Serious Mental Illness		No Serious Mental Illness	
	n	(%)	n	(%)	n	(%)
**Sample Size**	359,500	100.0	278,059	100.0	81,441	100.0
**Year**						
2013	45,093	12.5	30,956	11.1	14,137	17.4
2014	42,651	11.9	30,041	10.8	12,610	15.5
2015	33,304	9.3	26,239	9.4	7,065	8.7
2016	31,767	8.8	26,896	9.7	4,871	6.0
2017	38,184	10.6	29,922	10.8	8,262	10.1
2018	46,402	12.9	36,456	13.1	9,946	12.2
2019	44,412	12.4	35,566	12.8	8,846	10.9
2020	41,636	11.6	32,899	11.8	8,737	10.7
2021	36,051	10.0	29,084	10.5	6,967	8.6
**Age, in years**						
18–29	40,406	11.2	29,286	10.5	11,120	13.7
30–39	72,898	20.3	55,129	19.8	17,769	21.8
40–49	101,831	28.3	78,810	28.3	23,021	28.3
50–59	113,521	31.6	90,570	32.6	22,951	28.2
60 and older	30,844	8.6	24,264	8.7	6,580	8.1
**Gender**						
Men	184,312	51.3	140,789	50.6	43,523	53.4
Women	174,595	48.6	136,910	49.2	37,685	46.3
Missing	593	0.2	360	0.1	233	0.3
**Education level**						
Special education	547	0.2	474	0.2	73	0.1
0–8th grade	16,764	4.7	14,084	5.1	2,680	3.3
9th to 11th grade	54,733	15.2	46,008	16.5	8,725	10.7
12th grade or GED^1^	98,776	27.5	78,994	28.4	19,782	24.3
More than 12th grade	39,694	11.0	31,644	11.4	8,050	9.9
Missing	148,986	41.4	106,855	38.4	42,131	51.7
**Employment status**						
Employed^2^	33,189	9.2	26,062	9.4	7,127	8.8
Unemployed	72,723	20.2	57,378	20.6	15,345	18.8
Not in the labor force	108,366	30.1	97,036	34.9	11,330	13.9
Missing	145,222	40.4	97,583	35.1	47,639	58.5
**Living arrangements**						
Homeless	27,509	7.7	23,340	8.4	4,169	5.1
Private residence	196,208	54.6	158,674	57.1	37,534	46.1
Other living arrangement	39,941	11.1	33,476	12.0	6,465	7.9
Missing	95,842	26.7	62,569	22.5	33,273	40.9
**Marital status**						
Never married	138,709	38.6	107,941	38.8	30,768	37.8
Married	23,620	6.6	17,580	6.3	6,040	7.4
Separated	18,935	5.3	15,448	5.6	3,487	4.3
Divorced, widowed	43,113	12.0	36,327	13.1	6,786	8.3
Missing	135,123	37.6	100,763	36.2	34,360	42.2
**Race and ethnicity**						
Black or African American	149,080	41.5	116,911	42.0	32,169	39.5
Hispanic or Latino of any race	32,552	9.1	26,959	9.7	5,593	6.9
White	126,105	35.1	93,861	33.8	32,244	39.6
Another race or ethnicity	13,297	3.7	10,364	3.7	2,933	3.6
Missing	38,466	10.7	29,964	10.8	8,502	10.4
**Number of Mental Health Diagnoses**						
One	181,655	50.5	130,728	47.0	50,927	62.5
Two	130,374	36.3	104,852	37.7	25,522	31.3
Three	47,471	13.2	42,479	15.3	4,992	6.1
**Mental Health Disorder Diagnosis**						
Anxiety Disorder Diagnosis	52,149	14.5	40,364	14.5	11,785	14.5
ADHD Diagnosis	6,412	1.8	4,465	1.6	1,947	2.4
Bipolar Disorder Diagnosis	94,591	26.3	82,191	29.6	12,400	15.2
Conduct Disorder Diagnosis	739	0.2	521	0.2	218	0.3
Delirium/Dementia Diagnosis	1,320	0.4	1,053	0.4	267	0.3
Depressive Disorder Diagnosis	115,347	32.1	93,901	33.8	21,446	26.3
Oppositional Defiant Disorder Diagnosis	256	0.1	179	0.1	77	0.1
Personality Disorder Diagnosis	30,033	8.4	26,713	9.6	3,320	4.1
Pervasive Developmental Disorder	160	0.0	123	0.0	37	0.0
Schizophrenia or other Psychotic Disorder Diagnosis	86,298	24.0	81,498	29.3	4,800	5.9
Trauma- or Stressor-Related Disorder Diagnosis	59,571	16.6	45,021	16.2	14,550	17.9
Other Mental Health Disorder Diagnosis	47,146	13.1	35,069	12.6	12,077	14.8

Percentages are column percentages

^1^General Education Development

^2^Employed: Full Time or Part Time is not differentiated

**Fig 1:**
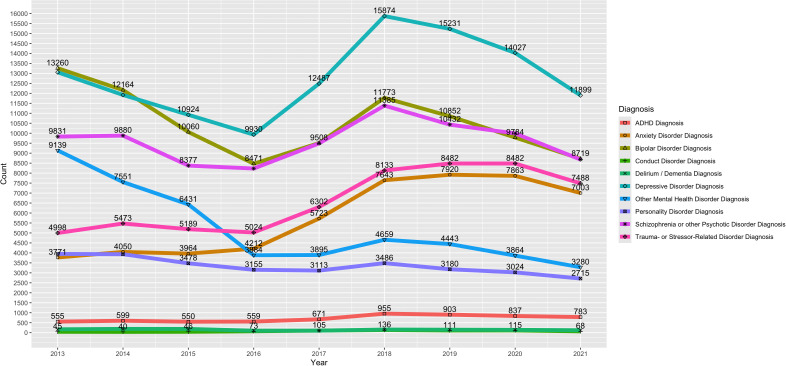
Trends of Mental Health Disorder Diagnoses Using Annual Count Data. Among Adults with a Cocaine Use Disorder Receiving Treatment from a Mental Health Facility.

The most endorsed mental health disorder diagnoses in the sample include depressive disorders (n = 115,347; 32.1%), bipolar disorders (n = 94,591; 26.3%), and schizophrenia or other psychotic disorders (n = 86,298; 24.0%). Fig 1 shows the trends of the mental health disorder diagnoses across the nine-year study period. Oppositional defiant disorder and pervasive developmental disorder were removed from Fig 1 due to small cell counts less than n < 20 for some years. Further, due to some lines overlapping in Fig 1, the count data are presented in Supplemental Table 1 (S1 Table).

### Serious mental illness

**Fig 2:**
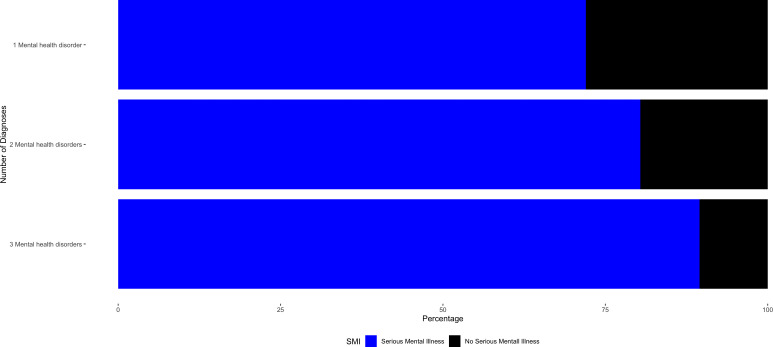
Percentage of Serious Mental Illness in each Number of Diagnoses Category. Among Adults with a Cocaine Use Disorder Receiving Treatment from a Mental Health Facility.

The majority of the sample was identified as having an SMI in the dataset (n = 278,059; 77.3%). Fig 2 shows the percentage of serious mental illness within each of the three number of mental health disorder categories.

**Fig 3:**
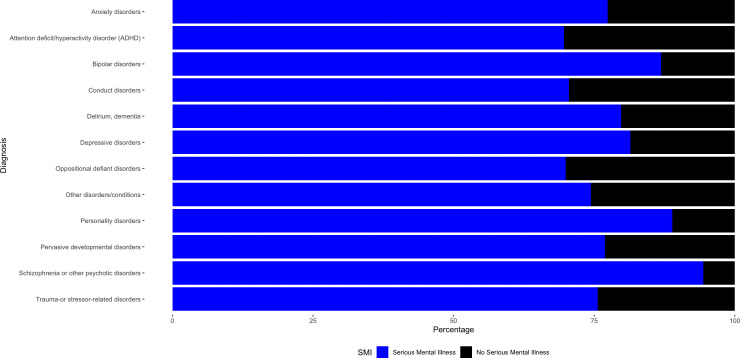
Percentage of Serious Mental Illness in each Mental Health Disorder Diagnosis Category. Among Adults with a Cocaine Use Disorder Receiving Treatment from a Mental Health Facility.

Fig 3 shows the percentage of serious mental illness within each mental health disorder diagnosis category. Data used to create Figs 2 and 3 may be found in Supplemental Tables 2 and 3 (S2 Table and S3 Table), respectively.

Results from the binary logistic regression models may be found in Table 2. While the results of the unadjusted binary logistic regression models may be found in the left portion of Table 2, we will interpret the results of the full model that is adjusted for each disorder. As seen in Table 2, anxiety disorders, bipolar disorders, conduct disorders, dementia/delirium, depressive disorders, personality disorders, pervasive developmental disorders, schizophrenia or other psychotic disorders, trauma-or stressor-related disorders, and other mental health disorders were associated with SMI. The largest associations were identified with schizophrenia or other psychotic disorders (Adjusted odds ratio (AOR)=20.188; 95% Confidence Interval (CI) = 19.533, 20.865), bipolar disorders (AOR = 7.257; 95%CI = 7.085, 7.433), and depressive disorders (AOR = 4.855; 95%CI = 4.754, 4.957). ADHD was associated with lower odds of SMI (AOR = 0.913; 95%CI = 0.860, 0.969).

**Table 2:** Mental health disorders associated with the presence of a serious mental illness.

Variable	Unadjusted Logistic Regression Models			Adjusted Logistic Regression Model		
	Odds Ratio	p-value	95% Confidence Interval	Odds Ratio	p-value	95% Confidence Interval
Anxiety disorder Diagnosis (Ref: No)	1.004	.744	0.982, 1.026	1.354	<.001***	1.322, 1.388
Attention deficit/hyperactivity disorder Diagnosis (Ref: No)	0.666	<.001***	0.631, 0.703	0.913	.003**	0.860, 0.969
Bipolar disorder Diagnosis (Ref: No)	2.336	<.001***	2.288, 2.385	7.257	<.001***	7.085, 7.433
Conduct disorder Diagnosis (Ref: No)	0.699	<.001***	0.597, 0.819	1.341	.001**	1.125, 1.599
Delirium, dementia Diagnosis (Ref: No)	1.156	.035*	1.010, 1.322	1.392	<.001***	1.197, 1.620
Depressive disorder Diagnosis (Ref: No)	1.426	<.001***	1.402, 1.452	4.855	<.001***	4.754, 4.957
Oppositional Defiant disorder Diagnosis (Ref: No)	0.681	.005**	0.521, 0.889	1.176	.285	0.874, 1.583
Personality disorder Diagnosis (Ref: No)	2.501	<.001***	2.410, 2.595	2.810	<.001***	2.701, 2.923
Pervasive Developmental Disorder Diagnosis (Ref: No)	0.974	.887	0.674, 1.406	1.781	.005**	1.193, 2.659
Schizophrenia or other Psychotic Disorder Diagnosis (Ref: No)	6.620	<.001***	6.423, 6.824	20.188	<.001***	19.533, 20.865
Trauma-or Stressor-Related Disorder Diagnosis (Ref: No)	0.888	<.001***	0.870, 0.907	1.469	<.001***	1.436, 1.504
Other Mental Health Disorder Diagnosis (Ref: No)	0.829	<.001***	0.811, 0.848	1.328	<.001***	1.295, 1.362

Ref: Reference Group

* < .05

** < .01

*** < .001

## Discussion

We examined the presence of mental health disorder diagnoses and SMI among a large national sample of adults with a cocaine use disorder who received treatment in mental health facilities from 2013 to 2021. This study identified [1] the most diagnosed mental health disorders, [2] the percentage of SMI, and [3] the associations between mental health diagnoses and SMI in this sample of adults with cocaine use disorder.

Depressive disorders, bipolar disorders, and schizophrenia or other psychotic disorders were the three most commonly diagnosed mental health disorders in this sample. Related to depressive disorders, Anhedonia, a potential symptom of cocaine use disorder, is associated with a loss of pleasure or a loss of interest in pleasure and activities that an individual used to enjoy [27,28]. Anhedonia is also a symptom of depressive disorders [5], and anhedonia is notable for higher clinical severity among substance use disorders co-occurring with depressive disorders [29]. This is worth noting as cocaine-induced depressive disorder (cocaine use that precipitates the development of a depressive disorder) [5] is likely impacted by the symptom anhedonia [29]. Considering that cocaine use disorder [30] and depressive disorders are associated with suicidal ideation [31] it is imperative to be prepared to treat both conditions should they co-occur. Further, targeting anhedonia, a symptom that could be shared between cocaine use disorder and depressive disorders, has the potential to improve treatment outcomes [32].

Bipolar disorders were the second most prevalent diagnosis in this sample and is known to co-occur with substance use disorders such as cocaine use disorder [33,34]. A prospective cohort study found that the lifetime use of cocaine was associated with major depressive disorder (a depressive disorder) converting to a bipolar disorder [33]. This may be expected since bipolar disorders are characterized by manic and depressed episodes. Also, cognitive impairment is associated with both bipolar disorders and cocaine use disorder and should be addressed in the treatment of either or both conditions [34,35]. Among hospitalized individuals with bipolar disorder, cocaine use is associated with not complying with medication regimens, further highlighting the importance of addressing both conditions simultaneously to improve treatment outcomes [36].

Schizophrenia or other psychotic disorders was the third most prevalent diagnosis in this sample. Cocaine use is one of the most commonly reported substances among individuals with schizophrenia [37]. Due to the high prevalence of substance use disorders among those with schizophrenia or other psychotic disorders [38–40], there is concern about worsening clinical profiles because of the psychostimulant effect of cocaine. It may be difficult to distinguish the effect of cocaine use disorder on the cognitive functioning of individuals with co-occurring schizophrenia or other psychotic disorders and cocaine use disorder.

Over three-fourths of the sample had an SMI based on state level definitions of SMI. Although most individuals in the sample had an SMI, it is imperative to consider the variability in definitions [18,19]. A systematic review examining the reliability of the term SMI found that approximately 26% of studies defined SMI as encompassing specific conditions such as bipolar and schizophrenia disorders [18]. Further, that systematic review found that the conditions with the highest prevalence among study samples include schizophrenia at 62%, bipolar disorders at 52%, and depressive disorders at 34% [18]. The results of this current study’s adjusted model follow this order, with the highest odds ratios being identified among individuals with a schizophrenia or other psychotic disorder diagnosis, a bipolar disorder diagnosis, and then a depressive disorder diagnosis.

Considering the data rely on state definitions of SMI, there could be vast variability in whether someone with cocaine use disorder and a co-occurring mental health disorder is documented as having an SMI or not. Essentially, crossing a state line to receive treatment from one facility to another could result in an individual not having a documented SMI to having an SMI and vice versa. Future policy analyses are necessary to identify potential differences and ranges in how SMI is defined across various states. Furthermore, such policy analyses may identify the potential for seeking uniformity in defining SMI across these states to achieve a more cohesive national mental healthcare system. Future studies are also needed to examine the healthcare, social, and political impacts of the co-occurrence of cocaine use disorder and SMI. Findings from this current study appear to suggest that most of the sample received treatment in a state that uses specific conditions (i.e., schizophrenia or other psychotic disorders) as guiding the definition of SMI. However, future national studies are needed that examine whether functional impairment is assessed when SMI is designated for individuals receiving treatment. Overall, this study provides a snapshot of the recent landscape of mental health disorders and SMI among individuals with a cocaine use disorder who received treatment from mental health facilities from 2013 to 2021. Significant comorbidity was identified, including multiple diagnoses and high rates of meeting state-level definitions of SMI.

### Limitation

Study limitations should be considered. One limitation is that some of the diagnoses were based on diagnostic groups from previous editions of the Diagnostic and Statistical Manual, such as delirium/ dementia diagnosis instead of neurocognitive disorders as found in the most recent edition Diagnostic and Statistical Manual of Mental Disorders, Fifth Edition, Text Revision released in 2022 [5]. Another limitation is that we are unable to assess the severity of diagnoses in this sample. Approximately 50% of the sample is missing data on demographic characteristics, which profoundly limits the ability to interpret sample sociodemographic characteristics. Although this study focuses on associations between specific mental health diagnoses and the presence of SMI, being able to include sociodemographic characteristics in the larger sample would result in more robust findings. Another limitation is that the dataset does not include descriptions of each state’s definition of SMI. Other limitations inherent to the dataset have been described elsewhere by SAMHSA, such as the MH-CLD not representing the total demand of mental health disorder treatment in the US [21].

## Supporting information

S1 Table Annual Count Data for Figure 1. (DOCX)

S2 Table Number of Diagnoses and SMI for Figure 2. (DOCX)

S3 Table Mental Health Disorders and SMI for Figure 3. (DOCX)
