# Supplementary material for: Cocaine use disorder, mental health diagnoses, and serious mental illness characteristics in mental health treatment
Source: PLOS Ment Health. 2026 Jan 22;3(1):e0000337. doi: 10.1371/journal.pmen.0000337 (PMC12826511; doi:10.1371/journal.pmen.0000337)
Supplement: S1 Table — (DOCX) [file pmen.0000337.s001.docx]

| **Supplemental Table 1. Annual Count Data for Figure 1** | | | | | | | | |  |  |  |  |
| --- | --- | --- | --- | --- | --- | --- | --- | --- | --- | --- | --- | --- |
| Year | Total Sample | SMI | Anxiety Disorder Diagnosis | ADHD Diagnosis | Bipolar Disorder Diagnosis | Conduct Disorder Diagnosis | Delirium / Dementia Diagnosis | Depressive Disorder Diagnosis | Personality Disorder Diagnosis | Schizophrenia or other Psychotic Disorder | Trauma- or Stressor-Related Disorder Diagnosis | Other Mental Health Disorder Diagnosis |
| 2013 | 45093 | 30956 | 3771 | 555 | 13260 | 45 | 169 | 13056 | 3948 | 9831 | 4998 | 9139 |
| 2014 | 42651 | 30041 | 4050 | 599 | 12164 | 40 | 190 | 11919 | 3934 | 9880 | 5473 | 7551 |
| 2015 | 33304 | 26239 | 3964 | 550 | 10060 | 46 | 187 | 10924 | 3478 | 8377 | 5189 | 6431 |
| 2016 | 31767 | 26896 | 4212 | 559 | 8471 | 73 | 100 | 9930 | 3155 | 8231 | 5024 | 3884 |
| 2017 | 38184 | 29922 | 5723 | 671 | 9508 | 105 | 103 | 12487 | 3113 | 9491 | 6302 | 3895 |
| 2018 | 46402 | 36456 | 7643 | 955 | 11773 | 136 | 157 | 15874 | 3486 | 11385 | 8133 | 4659 |
| 2019 | 44412 | 35566 | 7920 | 903 | 10852 | 111 | 150 | 15231 | 3180 | 10432 | 8482 | 4443 |
| 2020 | 41636 | 32899 | 7863 | 837 | 9784 | 115 | 142 | 14027 | 3024 | 9990 | 8482 | 3864 |
| 2021 | 36051 | 29084 | 7003 | 783 | 8719 | 68 | 122 | 11899 | 2715 | 8681 | 7488 | 3280 |
