# Supplementary material for: Cocaine use disorder, mental health diagnoses, and serious mental illness characteristics in mental health treatment
Source: PLOS Ment Health. 2026 Jan 22;3(1):e0000337. doi: 10.1371/journal.pmen.0000337 (PMC12826511; doi:10.1371/journal.pmen.0000337)
Supplement: S2 Table — (DOCX) [file pmen.0000337.s002.docx]

| **Supplemental Table 2. Number of Diagnoses and SMI for Figure 2** | |  |  |
| --- | --- | --- | --- |
| **Number of Diagnoses** | **SMI Status** | **Percentage** |  |
| 1 Mental health disorder | Serious Mental Illness | 72.0 |  |
| 1 Mental health disorder | No Serious Mental Illness | 28.0 |  |
| 2 Mental health disorders | Serious Mental Illness | 80.4 |  |
| 2 Mental health disorders | No Serious Mental Illness | 19.6 |  |
| 3 Mental health disorders | Serious Mental Illness | 89.5 |  |
| 3 Mental health disorders | No Serious Mental Illness | 10.5 |  |
|  |  |  |  |
|  |  |  |  |
