# Supplementary material for: Cocaine use disorder, mental health diagnoses, and serious mental illness characteristics in mental health treatment
Source: PLOS Ment Health. 2026 Jan 22;3(1):e0000337. doi: 10.1371/journal.pmen.0000337 (PMC12826511; doi:10.1371/journal.pmen.0000337)
Supplement: S3 Table — (DOCX) [file pmen.0000337.s003.docx]

| **Supplemental Table 3. Mental Health Disorders and SMI for Figure 3** | |  |
| --- | --- | --- |
| **Mental Health Disorder** | **SMI Status** | **Percentage** |
| Anxiety disorders | Serious Mental Illness | 77.4 |
| Anxiety disorders | No Serious Mental Illness | 22.6 |
| Attention deficit/hyperactivity disorder (ADHD) | Serious Mental Illness | 69.6 |
| Attention deficit/hyperactivity disorder (ADHD) | No Serious Mental Illness | 30.4 |
| Bipolar disorders | Serious Mental Illness | 86.9 |
| Bipolar disorders | No Serious Mental Illness | 13.1 |
| Conduct disorders | Serious Mental Illness | 70.5 |
| Conduct disorders | No Serious Mental Illness | 29.5 |
| Delirium, dementia | Serious Mental Illness | 79.8 |
| Delirium, dementia | No Serious Mental Illness | 20.2 |
| Depressive disorders | Serious Mental Illness | 81.4 |
| Depressive disorders | No Serious Mental Illness | 18.6 |
| Oppositional defiant disorders | Serious Mental Illness | 69.9 |
| Oppositional defiant disorders | No Serious Mental Illness | 30.1 |
| Personality disorders | Serious Mental Illness | 88.9 |
| Personality disorders | No Serious Mental Illness | 11.1 |
| Pervasive developmental disorders | Serious Mental Illness | 76.9 |
| Pervasive developmental disorders | No Serious Mental Illness | 23.1 |
| Schizophrenia or other psychotic disorders | Serious Mental Illness | 94.4 |
| Schizophrenia or other psychotic disorders | No Serious Mental Illness | 5.6 |
| Trauma-or stressor-related disorders | Serious Mental Illness | 75.6 |
| Trauma-or stressor-related disorders | No Serious Mental Illness | 24.4 |
| Other disorders/conditions | Serious Mental Illness | 74.4 |
| Other disorders/conditions | No Serious Mental Illness | 25.6 |
